# Molecular Docking of Aromatase Inhibitors

**DOI:** 10.3390/molecules16053597

**Published:** 2011-04-28

**Authors:** Naravut Suvannang, Chanin Nantasenamat, Chartchalerm Isarankura-Na-Ayudhya, Virapong Prachayasittikul

**Affiliations:** 1 Center of Data Mining and Biomedical Informatics, Faculty of Medical Technology, Mahidol University, Bangkok 10700, Thailand; 2 Department of Clinical Microbiology and Applied Technology, Faculty of Medical Technology, Mahidol University, Bangkok 10700, Thailand

**Keywords:** aromatase, aromatase inhibitors, molecular docking, drug design

## Abstract

Aromatase is an enzyme that plays a critical role in the development of estrogen receptor positive breast cancer. As aromatase catalyzes the aromatization of androstenedione to estrone, a naturally occurring estrogen, it is a promising drug target for therapeutic management. The undesirable effects found in aromatase inhibitors (AIs) that are in clinical use necessitate the discovery of novel AIs with higher selectivity, less toxicity and improving potency. In this study, we elucidate the binding mode of all three generations of AI drugs to the crystal structure of aromatase by means of molecular docking. It was demonstrated that the docking protocol could reliably reproduce the interaction of aromatase with its substrate with an RMSD of 1.350 Å. The docking study revealed that polar (D309, T310, S478 and M374), aromatic (F134, F221 and W224) and non-polar (A306, A307, V370, L372 and L477) residues were important for interacting with the AIs. The insights gained from the study herein have great potential for the design of novel AIs.

## 1. Introduction

Globally cancer is one of the leading causes of death, with an estimated 7.6 million deaths in 2007. In women, breast cancer is the leading cause of death and new cancer cases [1]. A common treatment for early-stage, hormone-sensitive breast cancer is surgery followed by radiotherapy. Furthermore, adjuvant endocrine therapy is given with or without chemotherapy, depending on tumor stage. In healthy women, estrogens are mainly produced in the ovaries and also in adipose tissue, breast, skin and bone [2]. Post-menopause, breasts are the major source of estrogen production. For the latter, the level of estrogens produced in the breast are comparable to that produced in the ovaries by pre-menopausal women which is four to six times higher than those found in serum. Approximately 60% of pre-menopausal and 75% of post-menopausal cancers are hormone-dependent [3], implying that endogenous estrogens are essentially required for proliferation. Many drugs used for the treatment of estrogen receptor-positive breast cancer are mechanistically based on interfering with either estrogen production or estrogen action.

**Figure 1:**
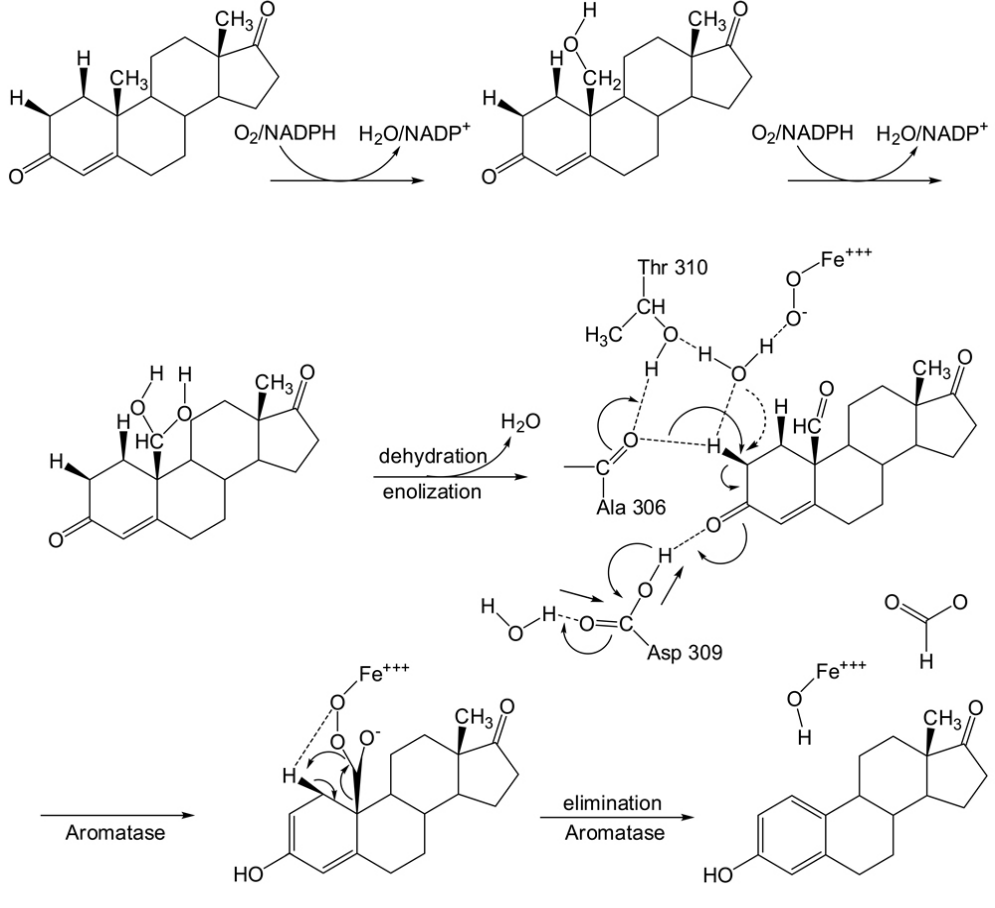
A possible mechanism for H1 abstraction [19], H2 abstraction and 2,3-enolization as proposed previously [20]. The catalysis starts with the hydroxylation of C19 of androstenedione to give C19-hydroxy-androstenedione by hydrogen abstraction–oxygen rebound mechanism. This is followed by another hydroxylation to the same carbon producing C19-dihydroxy-androstenedione after which dehydration yields C19-aldehyde-androstenedione. Afterwards, a nucleophilic attack on C2-H2 by the A306 CO-HO T310 pair takes place along with an electrophilic attack on the C3 carbonyl by a protonated D309 side chain. Finally, the electrons delocalize to form an aromatic system and estrone and formic acid are released as products. The arrows represent the direction of electron flow from the proton relay network. Involvement of a catalytic water molecule in H2 abstraction is a possibility. Dotted arrow shows the nucleophile of the backbone carbonyl from the A306 CO-OH T310 pair aided by a potential catalytic water molecule.

Cytochrome P450 19A1 (CYP19A1; EC 1.14.14.1), commonly known as aromatase, is an enzyme located in the endoplasmic reticulum of estrogen-producing cells that functions in the conversion of androgens to estrogens. It is comprised of a polypeptide chain of 503 amino-acid residues and a prosthetic heme group at its active site. An androgen-specific cleft consisting of hydrophobic and polar residues is situated within the confinement of the aromatase binding site. Such cleft is specific for androstenedione binding to catalyze androgen to estrogen via a three-step process. Each step requires one mol of O_2_, one mol of NADPH and NADPH cytochrome reductase. This reaction converts androstenedione, testosterone and 16α-hydroxytestosterone to estrone, 17β-estradiol and 17β,16α-estriol, respectively [4]. The two initial steps are the typical C19-methyl hydroxylation, while aromatization of the steroid A-ring is catalyzed at the final step (Figure 1). To block estrogen production, it is necessary to inhibit the enzyme through the use of aromatase inhibitors (AIs). AIs are only effective for post-menopausal women since they do not block estrogen production in the ovaries but act only on the local estrogen produced by breast cancer cells. Therefore, AIs serve as front-line therapy for estrogen-dependent breast cancer [1].

**Figure 2:**
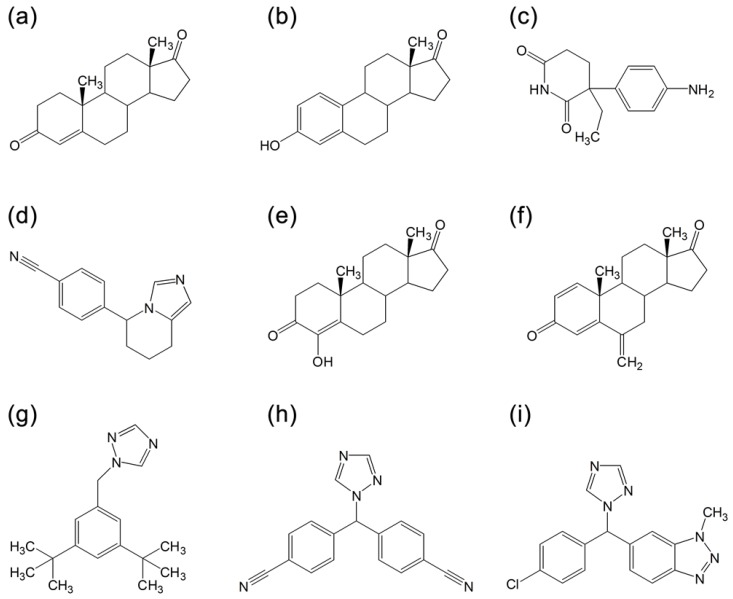
Structures of: **(a)** androstenedione, **(b)** estrone, **(c)** aminoglutethimide, **(d)** fadrozole, **(e)** formestane, **(f)** exemestane, **(g)** anastrozole, **(h)** letrozole and **(i)** vorozole.

To date, three generations of AIs are available. The first generation of AI is aminoglutethimide [5,6,7,8] (Figure 2) that was marketed in the late 1970s. Unfortunately, aminoglutethimide was far from being an ideal drug since it exhibited several drawbacks, most notably high toxicity [9,10] and lack of selectivity, since it can inhibit other CYP450 enzymes involved in cortisol and aldosterone biosynthesis [11]. Such flaws limited its use and led to its eventual withdrawal from the market. Nevertheless, aminoglutethimide served as the prototype for later AIs with emphasis on developing more potent, selective and less toxic AIs. Continuing on to the second generation, fadrozole (Figure 2), which contains an imidazole group [12], is more selective and potent than aminoglutethimide. Nevertheless, it still displayed effects on aldosterone, progesterone and corticosterone biosynthesis. Formestane [13] (Figure 2), a steroid analogue, was the first selective AI used in clinical trial. It was demonstrated to be effective and was well tolerated [14].

Finally, the third generation of AIs includes two triazole derivatives, anastrozole [15] (Figure 2) and letrozole [16] (Figure 2), and one steroid analogue, exemestane [16] (Figure 2). These AIs displayed improved efficacy and lower toxicity as compared with the estrogen antagonist, tamoxifen, in both early and advanced breast cancer [17,18]. For this reason, the last generation of AIs has been recommended by the FDA as first-line drugs for therapy of breast carcinoma. Anastrozole and letrozole, are non-steroid derivatives and competitive inhibitor of androstenedione.

Both contain a triazole group which interacts with the prosthetic heme group of aromatase. Exemestane is a steroidal analog that is catalytically converted into a chemically reactive species, leading to irreversible inactivation of aromatase. Mechanistically, the redox partner NADPH cytochrome reductase and cofactor NADPH are required. Furthermore, the inactivated enzyme can be finally eliminated by proteasomes [21].

Initial attempts to clarify the interaction mechanism of aromatase and its inhibitors have relied on the use of homology-derived models [22]. Such studies have focused on clinically used AIs such as fadrozole, letrozole and exemestane, as well as other natural products such as lignans, flavonoids and coumestrol [23,24,25,26]. Recently, the crystal structure of human placental aromatase has been solved by Ghosh *et al*. [20]. The availability of structural details on the active site of aromatase helps in understanding the binding characteristics of AIs as well as the evaluation of key reaction needed in the mechanism of aromatase. This opens up a plethora of opportunities by enabling the understanding of the molecular basis for the specificity of the aromatase enzyme and its unique catalytic mechanism, which is imperative for the development of the next-generation of AIs. Computational chemistry and relevant molecular simulation approaches have been successfully employed for studying the chemical reactions and binding mechanisms of a wide range of biological and chemical systems [27,28,29,30,31,32,33,34,35,36,37,38,39,40,41,42]. As such, *in silico* tools such as molecular docking are important for shedding light on the binding modalities and interaction strengths of AIs. Recently, Roy and Roy [43] performed molecular docking and three-dimensional quantitative structure-activity relationship study on a diverse set of compounds using the crystal structure of aromatase. To the best of our knowledge, the binding mode of all three generations of AI drugs within the crystal structure of aromatase has never been explored.

So far, numerous computational tools are available for drug design, and molecular docking provides an excellent platform for understanding the molecular mechanism governing enzyme inhibition. In this study, we elucidate the binding mode of aromatase with all three generation of AIs by means of molecular docking to the crystal structure of aromatase. Furthermore, the molecular conformation of each AIs was geometrically optimized at the B3LYP/6-31G(d) level while the protein structure of aromatase was subjected to molecular dynamics simulation. It is anticipated that the information gained from this study will be beneficial for further design of novel AIs.

## 2. Results and Discussion

### 2.1. Analyzing the Binding Site of Aromatase

Analysis using PDBsum [44] revealed that the binding site of aromatase occupies an inner cavity volume of 1525.92 Å^3^. Furthermore, entrance to the binding site as measured using PyMOL was found to be 3.24 Å in diameter. It is postulated that bulkier compounds may find it difficult to enter the binding site as it may need to rotate its side chains to accommodate access to the cavity entrance. On the other hand, small, rigid and less bulky compounds may find it easier to gain access to the binding site as it could readily diffuse in. Traditional approaches to AI development focus on inhibiting aromatase by binding to its active site. It may be worthwhile to consider the development of drugs that may sterically block access of natural substrate to the entrance of the binding site.

### 2.2. Molecular Dynamics Studies

The conformational flexibility of a protein’s binding site is an important issue in molecular docking studies. Previous work by Ni *et al*. [45] and Becker *et al*. [46] suggested that side chains in the binding site of apo and holo (inhibitor-bound) enzyme exhibited profound differences. This can be attributed to the fact that proteins display significant structural rearrangement of up to 7 Å backbone RMSD upon ligand binding [47]. Therefore, to overcome the issue of conformational flexibility of the protein binding site, substrate-bound aromatase (PDB id 3EQM) was used as a docking target. As the crystal structure of the protein is rather strained in terms of its conformation, molecular dynamics simulation was performed to allow the protein structure to relax to its equilibrium conformation. The effects that molecular dynamics simulation has on molecular docking, was investigated by using the structure of aromatase obtained directly from the Protein Data Bank and the equilibrium structure derived from molecular dynamics simulation.

**Figure 3:**
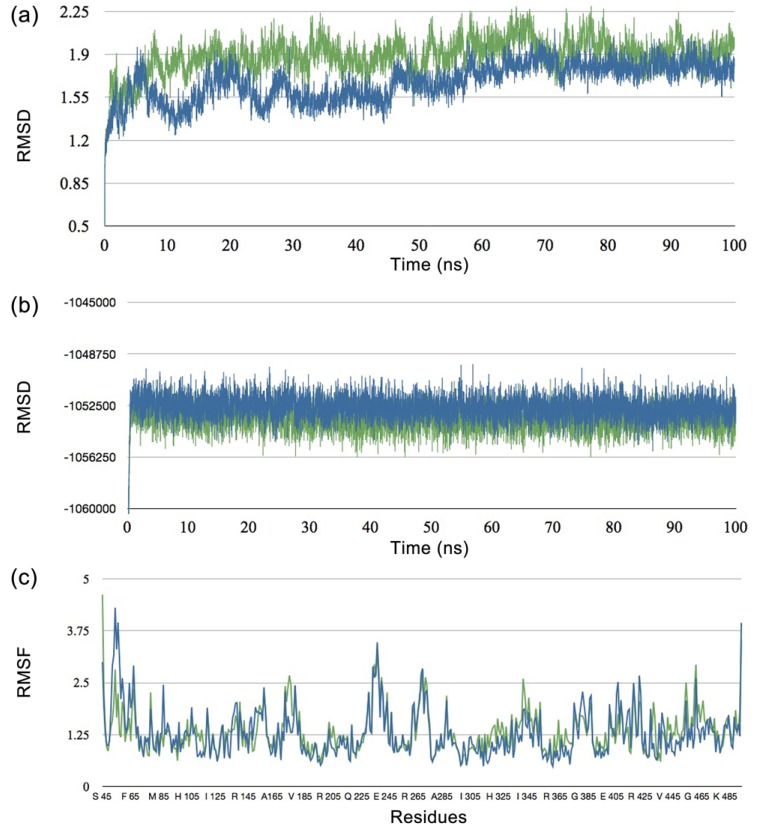
Result from molecular dynamics simulation showing the (**a**) RMSD, (**b**) Energy and (**c**) RMSF of aromatase in its bound (blue) and unbound (green) form.

**Figure 4:**
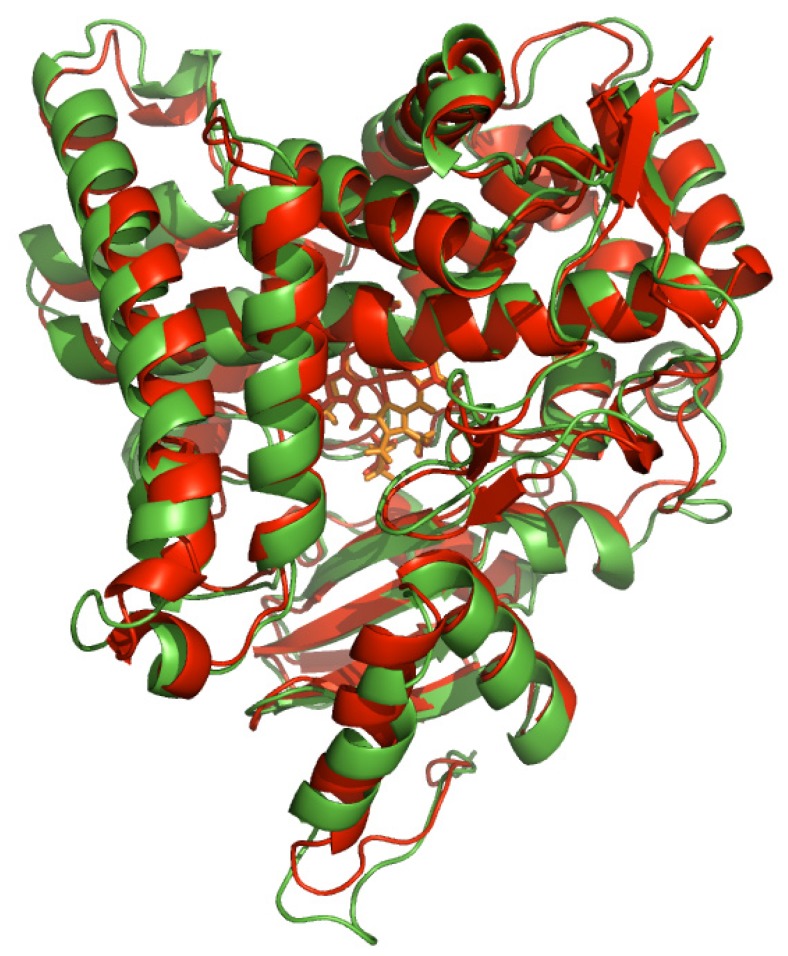
The structural alignment of aromatase at 0 ns (red) and 100 ns (green).

Figure 3 shows that there is significant structural rearrangement in the early phase of molecular dynamics simulation while gradually reaching equilibrium after 70 ns. In this study, the equilibrium structure from molecular dynamics simulation at 100 ns, which shows minimal fluctuation in structural RMSD, was selected for molecular docking investigations. Results on the flexibility of catalytic residues T310, M374 and S478 over time indicated that there is minimal fluctuation as observed by low RMSF values of 0.790, 0.840 and 0.827, respectively (Figure 3c). This suggests that such residues are quite rigid and therefore a rigid receptor docking approach is sufficient. The protein structures before and after molecular dynamics simulation was then compared by superimposing the structures (Figure 4). It is observed that the two structures displayed an RMSD of 1.944.

### 2.3. Docking Studies

**Figure 5:**
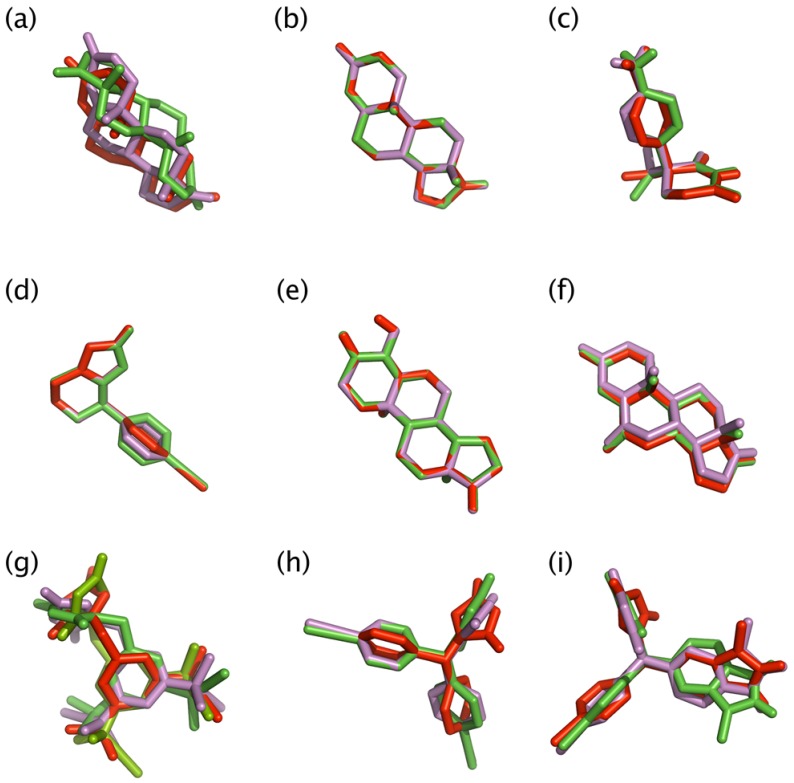
Superimposition of crystallized structure of androstenedione (shown as red sticks), docked conformer of aromatase at 0 ns of MD simulation (shown as purple sticks) and docked conformer of aromatase at 100 ns of MD simulation (shown as green sticks) poses of androstenedione **(a)** for validating docking protocol. Structure superimposition of **(b)** androstenedione, **(c)** aminoglutethimide, **(d)** fadrozole, **(e)** formestane, **(f)** exemestane, **(g)** anastrozole, **(h)** letrozole and **(i)** vorozole for elucidating the effect of MD simulation.

Molecular docking was performed to elucidate the binding mode of aromatase and its inhibitors. To this end, a total of seven AIs, one substrate and one product (Figure 2) were docked to the aromatase binding site. AutoDockTools [48] was used to prepare the molecules and parameters before submitting it for docking analysis with AutoDock and PyRx. To evaluate the validity of the docking system, the bound substrate was removed from the crystallized structure of aromatase and re-docked to the enzyme (Figure 5a). Results indicated that the X-ray crystallography conformer was nearly identical to the docked conformer, as deduced from superimposition of the two structures that displayed an RMSD of 1.350 Å. Moreover, the superimposed binding pose of androstenedione shown in Figure 5b are similar to those reported previously for both X-ray crystallographic structure [20] and homology models [49,50].

Binding pose with the lowest docked energy belonging to the top-ranked cluster was selected as the final model for post-docking analysis with AutoDockTools and PyMOL. The docking poses for androstenedione, fadrozole, formestane and exemestane displayed a single mode of ligand-receptor interaction as observed by the presence of a single frequency bar in the clustering histogram. On other hand, the docking poses for aminoglutethimide, anastrozole, letrozole and vorozole displayed multiple frequency bars and the most populated one with the lowest energy was selected for further investigation. Results obtained from AutoDock provided pertinent information on the binding orientation of ligand-receptor interactions that will be discussed in the next section. Furthermore, the free energies of binding (Δ*G*_b_) and inhibition constants (*K*_i_) as calculated by AutoDock are summarized in Table 1 along with their corresponding experimental inhibition concentration (IC_50_) [13].

**Table 1:** The approximate free energies of binding (Δ*G*_b_) and inhibition constants (*K*_i_) calculated by AutoDock along with their experimental inhibition concentration (IC_50_).

Ligands	Number of rotational bonds	Binding Energy(Δ*G_b_*) (kcal/mol)		Inhibition constant(*K_i_*) (nM)	IC_50 _(nM)
		Cluster	Best Energy		
Androstenedione ^a^	0	100	−11.04	8.06	
Aminoglutethimide ^b^	3	71	−7.85	1750.00	20000
	3	23	−7.65	2480.00	20000
Fadrozole ^c^	1	61	−7.95	1490.00	30
	1	39	−8.18	1010.00	30
Formestane^ c^	1	100	−10.99	8.77	30
Exemestane ^c^	0	100	−11.30	5.24	15
Anastrozole ^d^					
major cluster	4	45	−9.09	218.04	8
minor cluster	4	38	−9.32	147.55	8
Letrozole^ d^	3	99	−8.78	367.20	2
Vorozole^ d^	3	83	−8.64	462.16	
	3	17	−8.66	451.26	
Estrone^ e^	1	100	−10.30	28.39	

^a^ natural substrate; ^b^ 1^st^ generation aromatase inhibitor; ^c^ 2^nd^ generation aromatase inhibitor; ^d^ 3^rd^ generation aromatase inhibitor; ^e^ product

### 2.4. Analyzing the Docking Results of Aromatase Inhibitors

In comparison of the three generations of AI development, it is observed that the inhibition potency in terms of IC_50_ had steadily increased from the first through the third generation (Table 1). This fact is supported by the good correlation that exists between ΔG_b_ and IC_50_ (r = 0.6283) as well as between K_i_ and IC_50_ (r = 0.7006) for the first two generations of AIs (aminoglutethimide, fadrozole, formestane and exemestane). On the other hand, no significant correlation exists between the aforementioned parameters for the remaining AIs (anastrozole and letrozole) in the third generation. However, a major contribution to the enhanced therapeutic efficiency observed in the third generation can be attributed to the fact that these AIs possessed longer half-life and lower toxicity [51,52,53]. It should be noted that scoring functions provide only an estimate of binding free energy and that several approximations has been made in its calculation therefore the results of which must be considered with care.

To discern the relationship between the various physicochemical properties of the AIs with its respective biological activity, the Pearson’s correlation coefficient (r) was computed and used for constructing an intercorrelation matrix. Based on this matrix, it was observed that IC_50_ was well correlated with Q_m_ and logP with r = 0.907 and −0.816, respectively (Table 2). Particularly, it can be deduced that low Q_m_ and high logP values were essential for good therapeutic activity, suggesting that effective AIs should not be too polar and should be relatively hydrophobic, respectively. Similarly, ΔG_b_ displayed good correlation with logP (r = −0.782), suggesting that high logP values or high lipophilicity was important for effective binding to aromatase (except anastrazole and exemestane). Similar to the trend that was observed for IC_50_, the Q_m_ (r = 0.871) and logP (r = −0.854) was also found to be positively and negatively correlated with K_i_.

**Table 2:** Summary of physicochemical descriptors of aromatase inhibitors.

	Q_m_	Energy (kcal/mol)	*μ*(debye)	HOMO(eV)	LUMO(eV)	HOMO-LUMO_gap_(eV)	RMSD	Log P
Androstenedione	0.211	-890.133	3.746	−0.232	−0.046	0.186	0	2.7
Aminoglutethimide	0.280	-765.007	3.626	−0.206	−0.025	0.181	1.04	1.2
Fadrozole	0.202	-705.565	5.687	−0.218	−0.061	0.157	0.16	2.1
Formestane	0.224	-965.357	2.178	−0.223	−0.053	0.169	0.08	2.6
Exemestane	0.206	-926.993	4.430	−0.235	−0.062	0.173	0	3.1
Anastrozole	0.232	-932.972	4.982	−0.265	−0.036	0.229	1.43	2.1
Letrozole	0.201	-928.146	3.911	−0.269	−0.073	0.196	1	2.7
Vorozole	0.200	-1446.710	1.871	−0.244	−0.051	0.193	0.26	3.1
Estrone	0.212	-849.592	3.981	−0.210	−0.015	0.195	0	3.1

**Figure 6:**
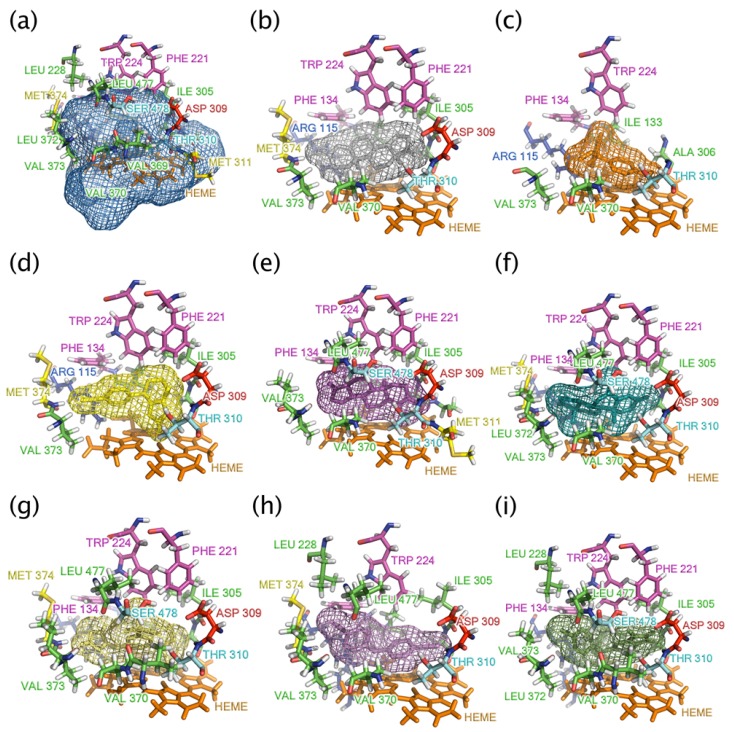
**(a)** The binding site of aromatase, represented as a mesh, is shown interacting with its natural substrate **(b)** androstenedione the following aromatase inhibitors **(c)** aminoglutethimide, **(d)** fadrozole, **(e)** formestane, **(f)** exemestane, **(g)** anastrozole, **(h)** letrozole and **(i)** vorozole. Environmental residues within a radius of 4 Å around the ligands are color-coded according to its molecular property as shown in green (non-polar), purple (aromatic), red (negatively-charged), cyan (polar), yellow (methionine) and orange (heme).

Table 3 summarizes the amino acid residues interacting with AIs. It is observed that aminoglutethimide (Figure 6c), the first generation of AI, engages in hydrophobic interaction with the aromatase binding site along with hydrogen-bond interactions with A306 and T310 [54]. In spite of having multiple points of interaction, surprisingly, aminoglutethimide had relatively low binding energy of −7.82 kcal/mol and rather high *K*_i_ of 2200 nM (Table 1). Such docking results corroborate its ineffectiveness as an AI as does the experimental results where the IC_50_ of aminoglutethimide is 20,000 nM [13].

The second generation of AI is composed of fadrozole (Figure 5d), formestane (Figure 5e) and exemestane (Figure 5f). It is observed that fadrozole and formestane displayed similar level of therapeutic efficiency (IC_50_ of 30 nM) but significant difference in the calculated Δ*G*_b_ (−8.88 and −13.35, respectively) and *K*_i_ (309.15 and 0.16, respectively). This can be attributed to the fact that fadrozole and formestane have different structural scaffold, in which the former is an azole derivative while the latter is a steroid derivative. Additionally, the docking poses for both structures were quite different in that fadrozole (Figure 5c) engages predominantly in hydrophobic van der Waals surface contact and coordinating its azole nitrogen to the heme iron atom, which contributed to a lower Δ*G*_b_ value (Table 1). Molecular docking simulations of proteins where ligand binding involves metal coordination (in our case the iron atom of the heme cofactor) poses a great challenge for molecular modeling. This issue has been addressed by researchers in several different ways. In studying aromatase inhibitors, a set of charges was obtained by Favia *et al*. for the heme cofactor and used in molecular docking and molecular dynamics simulations [55] while Neves *et al*. applied distance restraints was applied to ensure the coordination of heterocyclic nitrogen to the iron atom of heme [56]. In a relevant study, Röhrig *et al*. developed a protocol to parameterize a scoring function to facilitate molecular simulation of heme-containing proteins.

**Table 3:** Summary of residues interacting with the aromatase inhibitors.

Ligand	H-bond Interaction	Metal coordination	Hydrophobic Interaction
Androstenedione	M374, T310		R115, F134, F221, W224, I305, A306, D309,
			V370, V373, M374
Aminoglutethimide	A306, T310		R115, I133, F134, W224, V370, V373
Fadrozole		Azole-Heme	R115, F134, F221, W224, I305, A306, D309,
			T310, V373, M374
Formestane	A306, T310		I133, F134, F221, W224, I305, A307, D309,
			M311, V370, L372, V373, S478
Exemestane	R115, M374		F134, F221, W224, A306, D309, T310,
			V370, L372, V373, L477, S478
Anastrozole			
major cluster	L372	Azole-Heme	R115, I133, F134, F221, W224, I305, A306,D309, T310, V370, V373, M374, L477, S478
			
minor cluster		Azole-Heme	R115, I133, F134, F221, W224, I305, A306, D309, T310, V369, V370, R435, L477, S478
Letrozole			R115, I133, F134, W224, L228, I305, A306, D309, T310, V370, L372, V373, M374, R435, L477
Vorozole	I305, D309	Azole-Heme	R115, I133, F134, F221, W224, L228, A306,
			T310, V369, V370, L372, V373, R435, L477, S478
Estrone	M374		R115, F134, A306, T310, V370, L372, V373

On the other hand, formestane (Figure 6d) takes part in both hydrophobic and hydrogen bond (with backbone amine group of M374 and with carboxylic acid of D309) interactions, which accounted for its higher Δ*G*_b_ value. Exemestane displayed potent inhibition but binds irreversibly to aromatase [57]. Similar to formestane, exemestane engages in a hydrogen bond formation with the backbone amine of M374 along with hydrophobic interactions. Furthermore, a comparative analysis of exemestane (Figure 6f) with androstenedione (Figure 6b) revealed that the former provided an extra methylidene group at the C6 position which affords a tighter and stronger van der Waals contact with the surrounding protein residues [20]. It is also interesting to note that formestane and exemestane, which are the only AIs with steroid scaffold, yielded the highest Δ*G*_b_ (−13.35 and −12.31, respectively) and the best performing *K*_i_ (0.16 and 0.95, respectively) values.

The third generation of AI is made up of anastrozole (Figure 6g), letrozole (Figure 6h), and vorozole (Figure 6i). It should be noted that these AIs differed from the previous two generations (except for fadrozole) in that it is comprised of nitrogen atoms, which is responsible for coordinating with the heme group [36]. Anastrozole and letrozole form strong but reversible coordination to the heme group [50,57].

In therapeutic terms, this generation possessed a higher degree of specificity, are 100-3,000 times more active and 96% more efficient in inhibiting the In therapeutic terms, this generation possessed a higher degree of specificity, are 100-3,000 times more active and 96% more efficient in inhibiting the aromatization of androstenedione throughout the body than that of aminoglutethimide [57]. Experimental evidences revealed that letrozole provided higher therapeutic potential than that of anastrozole, which gave IC_50_ of 2 and 8, respectively.

Examination of the calculated quantum chemical properties also revealed that a less polar molecule is favorable for good therapeutic efficiency as letrozole provided a lower value than anastrozole for the following parameters: Q_m_ (0.201 and 0.232, respectively), *μ* (3.911 and 4.982, respectively) and logP (2.7 and 2.1, respectively). In spite of the higher interaction energy as afforded by letrozole, the clinically usage of anastrozole is more widespread owing to its lower side effects as compared to letrozole [13].

### 2.5. Key Interaction Residues

Table 3 summarizes the key interaction residues of aromatase with the ligands as revealed through molecular modeling analysis (Figure 6). Particularly, amino acid residues located within the vicinity of 4 Å from the ligands were shown and their prospective interaction types were inferred. It is observed that all AIs engage in hydrophobic interactions with the following residues: F134, W224 T310 and V373. A majority of AIs participate in hydrophobic interactions with F221, (except for aminogletethimide, letrozole), A306, (except for formestane), V370 (except for aminoglutethimide), L372 (except for fadrozole), and S478 (except for aminoglutethimide, fadrozole and letrozole). On the other hand, some residues such as L228 are found to interact with only letrozole and vorozole, A307 and M311 interacted with only formestane, I305 interacted with only formestane and exemestane, V369 interacted with only anastrozole minor cluster and vorozole, and R435 interacted with anastrozole minor cluster, letrozole and vorozole.

### 2.6. Analysis of Structure-activity Relationship

An analysis of the structure-activity relationship of AIs in relation to its IC_50_ values (Table 1) revealed that AIs containing heterocyclic nitrogen atoms gave the lowest IC_50_ values (letrozole and anastrozole having IC_50_ values of 2 and 8 nM, respectively) while AIs with a steroid scaffold gave slightly higher IC_50_ values (exemestane and formestane, with IC_50_ values of 15 and 30 nM, respectively). Furthermore, the results revealed that AIs with heterocyclic nitrogen atoms (letrozole and anastrozole) had higher numbers of rotatable bonds (letrozole and anastrozole had 3 and 4 rotatable bonds, respectively) while AIs with a steroid scaffold had fewer (exemestane and formestane had 0 and 1, respectively), which is to be expected as steroids generally have rigid structures while the former had rotatable bonds linking several heterocycles. It follows that when the number of rotatable bonds are high the IC_50_ values will be low (except for aminoglutethimide) that is <10 nM (letrozole and anastrozole had three and four rotatable bonds and an IC_50_ of 2 and 8 nM, respectively) while the reverse is true where fewer number of rotatable bonds gave higher IC_50_ values of >10 nM (exemestane, formestane and fadrozole had 0, 1 and 1 rotatable bonds and an IC_50_ of 15, 30 and 30 nM, respectively). Furthermore, Table 1 shows that AIs with heterocyclic nitrogen atoms displayed multiple cluster of docking conformation (aminoglutethimide, fadrozole, anastrozole and vorozole; except for letrozole) while androstenedione and steroidal AIs had a single cluster of docking conformation. Moreover, steroidal AIs possessed stronger level of binding energy (>−10.9 kcal/mol) than the nitrogen-containing heterocycles (−7.65 to −9.32 kcal/mol) which may be attributed to the van der Waals interaction of steroidal AIs with the binding cavity.

### 2.7. Mechanism of aromatase catalysis

The catalytic activity of aromatase is essentially a three-step process involving the conversion of androstenedione and testosterone to estrone and estradiol, respectively [4]. The first two steps entails the hydroxylation of C19-methyl group, which is modulated by three amino acid residues, comprising A306 and T310 [20], and two catalytic water molecules (Figure 1), which activates the ferrous dioxygen to the hydroxylating Fe(IV) = O form. This is followed by a H2β abstraction of the 2,3-enolization process in the aromatization step that essentially entails a nucleophilic attack on H2β-C by A306 and T310 along with concerted electrophilic attack on C3-keto oxygen by D309 to drive H2β abstraction and 2,3-enolization. Finally, the electrons delocalize to form an aromatic system and estrone and formic acid are released as products. As many cofactors (e.g., H_2_O, O_2_ and NADPH) are needed for catalytic activity, its proper entrance into the binding site is essential for catalytic activity. It then follows that decreasing the space within the binding cavity by means of ligand docking may hinder the entrance of cofactors into the cavity resulting in the inhibition of the aromatization process. The occupation of androstenedione within the aromatase binding site leaves a larger empty space within the cavity when compared to the empty space remaining after binding to AIs (Figure 6).

### 2.8. Comparing Between Substrate and Product of Aromatase Enzyme

In order to consider the releasing mechanism, the binding affinity of the substrate (androstenedione) were compared to the product (estrone) [4]. It is observed that Δ*G*_b_ for the substrate was higher than that of the products. Particularly, the conversion of androstenedione to estrone resulted in a decrease of Δ*G*_b_ from −10.09 to −10.30 kcal/mol (Table 1). Likewise, reductions in *K*_i_ values were also observed for androstenedione to estrone (10.15 to 28.39 nM) conversions. Such lower affinities to the aromatase binding site of the products favor its release from the cavity. It is also interesting to note that the energetic differences of the pre and post docked conformer of the substrates displayed negligible change whereas a higher magnitude of change was observed for the products, particularly favoring the free form (−849.6044 for androstenedione) over that of the docked form (−849.5918 for estrone). Such disparity may be attributed to the fact that the products interact weakly with the binding site and therefore give rise to a lower total energy of the molecule. As the free form of the products possessed a higher energetic level than its docked form, this suggests that the unbound form would be more energetically favorable for the products.

## 3. Experimental

### 3.1. Preparation of Protein and Ligand Structures

The crystal structure of aromatase (PDB id 3EQM) was used as a docking target for all three generations of AI drugs after removal of the natural substrate androstenedione. To prepare the aromatase structure for docking, essential hydrogen atoms, Kollman united atom charges and solvation parameters were added using AutoDockTools [48] and PyRx0.3 [58]. To account for metal coordination in performing docking simulations. A set of charge obtain from the work of Favia *et al*. [59] was applied on the heme residues and iron atom. The AI drugs, comprising aminoglutethimide, formestane, fadrozole, anastrozole, vorozole and letrozole. were obtained from PubChem (http://pubchem.ncbi.nlm.nih.gov) and used as ligands. The ligand structures were geometrically optimized with Gaussian 09W [60] using the B3LYP/6-31G(d) [61] method. Next, the ligand structures were prepared for docking by merging non-polar hydrogen atoms, adding Gasteiger partial charges and defining rotatable bonds.

### 3.2. Molecular dynamics simulation

As previously mentioned, the atomic coordinate of the protein was obtained from Protein Data Bank using PDB id 3EQM. The apo and holo forms of the protein were subjected to molecular dynamics simulation which was performed using YASARA, version 10.11.28 [62,63], with the AMBER03 force field [64]. The holo or androstenedione-bound form was used as is while the apo form was generated by removing androstenedione from the binding cavity. The protein was then placed in a water box that is 10 Å larger than each side of the protein. Hydrogen atoms were added to the protein structure at the appropriate ionizable groups according to the computed pKa in relation to the simulation pH, thus a hydrogen atom will be added if the computed pKa is higher than the pH. The pKa is computed for each residue according to the Ewald method [65]. The structure was then minimized using steepest-descent method followed by simulated annealing. The simulation was performed at pH 7.0 in a 0.9% NaCl solution at 300 K temperature for 100 ns. A cutoff of 7.86 Å was used for van der Waals forces while Particle Mesh Ewald algorithm [66] were used for electrostatic forces. A multiple time step of 1.25 and 2.5 fs. were used for intramolecular and intermolecular forces, respectively. All calculation were done on an Intel Core2Quad 2.66 GHz with 4 GB of RAM.

### 3.3. Molecular Docking

Docking calculations were performed with AutoDock, version 4.2 using the Lamarckian Genetic Algorithm [67] and PyRx 0.3 (http://pyrx.scripps.edu) [58]. A grid box size of 50 x 64 x 78 Å points with a grid spacing of 0.375 Å was generated using AutoGrid [48]. The grid was centered at x,y,z coordinates of 83.35, 49.60, 50.60, which was reported as the binding site residues [20]. AutoDock parameter set- and distance-dependent dielectric functions were used for calculating the van der Waals and the electrostatic terms, respectively. The initial position, orientation and torsions of the ligand molecules were set randomly. Each docked compound was derived from 100 independent docking runs that were set to terminate after a maximum of 2.5 × 10^6^ energy evaluations with mutation rate of 0.02 and crossover rate of 0.8. The population size was set to use 250 randomly placed individual. The search for low-energy binding orientations was performed by Lamarckian Genetic Algorithm using a translational step of 0.2 Å, a quaternion step of 5 Å and a torsion step of 5 Å. In order to validate the accuracy of the docking system, the nature substrate androstenedione was re-docked to aromatase and its orientation with respect to the crystal structure was determined. The root mean square deviation (RMSD) between the observed crystal structure and the predicted conformation obtained from AutoDock gave RMSD value of 1.350 Å (Figure 6a), indicating that the protocol was sound. The best docked conformations as deduced from the clustering histogram were those with low binding energy and highly populated cluster. Each bin of the clustering histogram is comprised of conformations within RMSD of 2 Å from its best docked conformation.

### 3.4. Post-docking analysis

Analysis of the docking results was performed using AutoDockTools [68] and PyMOL [69]. Such tools can help elucidate which type of interaction (e.g. hydrogen-bond, π-π interaction and cation-π interaction) contributed to ligand binding. The most favorable ligand binding poses as revealed by clustering histograms along with their corresponding binding energy was obtained from AutoDockTools. Additionally PyMOL were used to provide complementary information on ligand-receptor interaction. All molecular graphics were rendered and ray-traced using PyMOL, version 0.99. Comparative analysis of *in vacuo* and docked conformer of AI drugs was carried out by superimposing both structures followed by calculating their root mean squared deviation (RMSD) in order to distinguish any conformational change that may take place upon protein binding. The RMSD of the superimposed structures were calculated without pair-fitting in PyMOL. Additionally, the molecular properties of the optimized ligand structures were obtained from quantum chemical calculations using Gaussian 09W at the B3LYP/6-31G(d) level. These parameters include the following: total energy, mean absolute charge (Q_m_), dipole moment (*μ*), energy of the highest occupied molecular orbital (HOMO), energy of the lowest unoccupied molecular orbital (LUMO), and HOMO-LUMO_gap_. The logarithmic transformed values for the partition coefficients (logP) of the ligands were obtained from PubChem [70].

## 4. Conclusions

In this study, insights into the interaction of aromatase with its inhibitors were elucidated through the use of molecular docking. The equilibrium structure of aromatase from molecular dynamics was obtained after 100 ns of simulation. AutoDock implementing the Larmarckian genetic algorithm was used to model the interaction between aromatase and its inhibitors. The docking protocol could reliably reproduce the interaction of aromatase with its substrate as observed from the RMSD of 1.350 Å for the superimposed crystal and re-docked structures. Results revealed that polar (D309, T310, S478 and M374), aromatic (F134, F221 and W224) and non-polar (A306, A307, V370, L372 and L477) residues were important for interacting with the AIs. The results indicated that AIs based on the steroid scaffold (e.g. formestane and exemestane) exhibited higher Δ*G*_b_ than the azole scaffold (fadrozole, anastrozole, letrozole and vorozole) which is possible due to the high structural rigidity of the former as opposed to the latter which was structurally flexible. The knowledge acquired from this study has important implications for the design of novel. To further enhance the therapeutic efficiency of AIs, further structural modifications of existing scaffolds or discovery of new scaffold is a promising venue to explore.
